# Effect of Nanoclay Dispersion on the Properties of a Commercial Glass Ionomer Cement

**DOI:** 10.1155/2014/685389

**Published:** 2014-08-26

**Authors:** Muhammad A. Fareed, Artemis Stamboulis

**Affiliations:** ^1^School of Metallurgy and Materials, University of Birmingham, Edgbaston, Birmingham B15 2TT, UK; ^2^FMH College of Medicine and Dentistry, University of Health Sciences Lahore, Lahore 54000, Pakistan

## Abstract

*Objective.* The reinforcement effect of polymer-grade montmorillonite (PGV and PGN nanoclay) on Fuji-IX glass ionomer cement was investigated.* Materials and Method. *PGV and PGV nanoclays (2.0 wt%) were dispersed in the liquid portion of Fuji-IX. Fourier-transform infrared (FTIR) spectroscopy and gel permeation chromatography (GPC) were used to quantify acid-base reaction and the liquid portion of GIC. The mechanical properties (CS, DTS, FS, and *E*_*f*_) of cements (*n* = 20) were measured at 1 hour, 1 day, and 1 month. The microstructure was examined by cryo-SEM and TEM.* Results*. FTIR shows that the setting reaction involves the neutralisation of PAA by the glass powder which was linked with the formation of calcium and aluminium salt-complexes. The experimental GICs (C-V and C-N) exhibited mechanical properties in compliance to ISO standard requirement have higher values than Fuji-IX cement. There was no significant correlation of mechanical properties was found between C-V and C-N. The average Mw of Fuji-IX was 15,700 and the refractive index chromatogram peak area was 33,800. TEM observation confirmed that nanoclays were mostly exfoliated and dispersed in the matrix of GIC.* Conclusion*. The reinforcement of nanoclays in GICs may potentially produce cements with better mechanical properties without compromising the nature of polyacid neutralisation.

## 1. Introduction

Since the discovery of glass ionomer cement (GIC) by Wilson and Kent [1] the family of GICs have evolved into a diverse group of dental materials that include direct restoratives, luting cements, liners, bases, atraumatic restoratives, and pit and fissure sealants [2]. They are available as conventional and resin-modified products. The conventional GIC consists of fluoroaluminosilicate glass powder and an aqueous solution of poly(acrylic acid) (PAA) copolymer. The conventional GICs undergo a chemical acid-base reaction by mixing the powder and the liquid portion. The GICs are versatile dental restorative materials due to the several beneficial properties such as chemical adhesion to the tooth structure, biocompatibility, and release of fluoride [2, 3]. However, the low mechanical strength and early water sensitivity make them unsuitable for use in load-bearing areas of molar teeth [4]. The properties of a conventional GIC are influenced by the glass-powder and chemical composition of the polymer liquid. Various modifications and the developments of glass-powder and polymer liquid have been introduced to improve the mechanical properties of GICs by altering the chemistry [5].

The incorporation of small amount of montmorillonite (nanoclay) in the polymer matrix leads to a remarkable improvement of the mechanical, physical, and chemical properties of the resulting composite as compared to conventional materials [6]. Polymer-nanoclay composites involve the interaction of polymer matrix with the nanoplates of clay and are formed by the dispersion of low weight percentages of nanoclay into polymers. Montmorillonite (MMT) nanoclays have attracted much research interests over the past decade and are widely used for dispersion in polymers due to the high aspect ratio and the large interface of the polymer-nanoclay interaction [7]. The mechanical performance (stiffness and strength) was enhanced significantly with addition of small amount (0.5–5.0 wt%) of nanoclay in polymers [8]. The application of different types of nanoclays in dental restorative materials resulted in improved physical, mechanical, and adhesion properties [9, 10]. The exfoliation of nanoclays in the liquid portion of GIC is the foremost towards the development of nanoclay reinforced glass ionomer cements. In this study, the reinforcement effect of polymer grade montmorillonite was explored by dispersion in the liquid portion of a GIC and subsequent properties of a commercial conventional GIC (Fuji-IX, GC Co.).

## 2. Materials and Methods

### 2.1. Experimental Materials

Conventional glass ionomer cement Fuji-IX GP (GC Corporation, Tokyo Japan, batch number 0704281, shade A2) was used as a control group. Fuji-IX glass powder is mainly composed of fluoroaluminosilicate glass containing Si, Al, Sr, and Na. Fuji-IX liquid is composed of poly(acrylic acid), copolymers of carboxylic acid, and tartaric acid. Purified polymer-grade (PG) montmorillonite (PGV and PGN nanoclay) was obtained from Nanocor Inc. (Chicago, IL, USA). The PGV and PGN nanoclay consisted of silicates nanoplates of 1 nm thickness (lengths were up to 1000 nm) and were purified to a level greater than 98% montmorillonite by the manufacture. Nanoclays were dispersed in Fuji-IX liquid (FL) by the exfoliation-adsorption method as reported previously [11]. Briefly, PGV and PGN nanoclay (0.16 grams = 2.0 wt%) were weighed on a balance (TS4000, Ohaus, Pine Brook, NJ, USA) and were mixed in FL on a hot plate (Stable Temp Cole-Parmer IL, USA) using a magnetic-stirrer at 100 rpm for 24 hours at 75°C. The schematic presentation of polymer solutions prepared is shown in Table 1.

### 2.2. Cements Specimen Preparation

Cylindrical specimens (6 × 4 mm) for compressive strength (CS) test, disk-shaped specimens (2 × 4 mm) for diametral-tensile strength (DTS) test, and rectangular bar-shaped specimens (25 × 2 × 2 mm) for three-point bend flexural strength (FS) test were prepared. Cement specimens were fabricated at room temperature using split brass moulds. A PTFE dry-film spray (PR Mould release RS-7 Rocol Leeds, UK) was used to prevent cement adhesion to the mould. Fuji-IX glass powder and the corresponding liquid were mixed using a stainless-steel spatula on a paper mixing-pad with a powder to liquid ratio of 3.6 : 1 as recommended by the manufacturer (Table 1). The mould was slightly overfilled and gently pressed with an acetate-sheet and glass-slides within 60 seconds of the end of mixing. The mould was tightened using a C-shaped screw clamp and was stored in a desiccator maintained at 37.5°C temperature and at 95% humidity for one hour. The set specimens were removed from the mould after one hour and defective specimens were discarded. The cement specimens were conditioned in distilled water at 37.5°C for 1 hour, 1 day, and 1 month before mechanical testing.

### 2.3. Measurement of Mechanical Properties

Mechanical testing was performed on a screw-driven Instron machine (Model 5566, Instron Corporation, High Wycombe, UK) at a cross-head speed of 1.0 mm/min. Twenty specimens (*n* = 20) were prepared for each of the three glass ionomer cements for each storage time (1 hour, 1 day, and 1 month) to measure CS, DTS, and FS (Table 1). The CS was calculated from the equation CS = 4*P*/*πd* ^2^, where *P* is the maximum force applied at fracture and *d* is the diameter of the specimen [12]. The DTS was determined from the relationship; DTS = 2*P*/*π*DT, where *P* is load at fracture and *D* is the diameter and *T* is the thickness of the specimen [13]. The FS in three-point bending was obtained from the formula FS = 3FL/2*bh* ^2^, where* L* (20.0 mm) is the span between the two supports,* b* is the breadth, and *h* is the height of the specimen [14]. The flexural modulus (*E*_*f*_) was calculated from the data of three-point bend test at one-month storage time by drawing a tangent to the steepest initial straight-line portion of the load-deflection curve. *E*_*f*_ was calculated from the equation *E*_*f*_ = *l* ^3^ *m*/4*bd* ^3^, where *E*_*f*_ is the modulus of elasticity in bending, *l* is the distance between the two supports,* m* is the slope of the tangent to the initial straight-line portion of the load-deflection curve N/mm of deflection, *b* is the width, and *d* is the depth (height) of the bar shaped specimen [15].

### 2.4. Working Time and Setting Time

**Figure 1:**
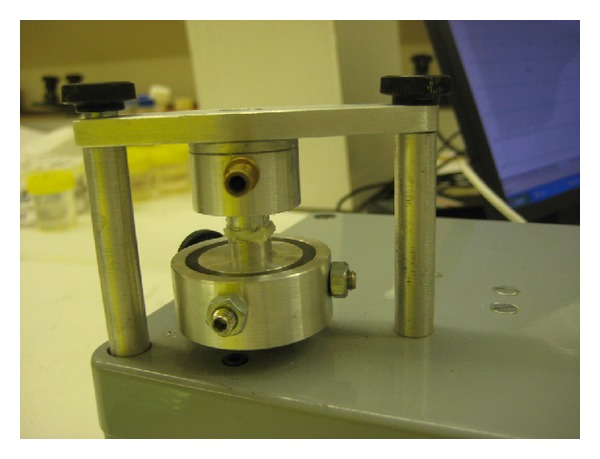
The custom made Wilson's oscillating rheometer.

The working time (WT) and the setting times (ST) of the cements were determined at an ambient temperature (21–25°C) by a modified Wilson oscillating rheometer shown in Figure 1. The oscillating rheometer consisted of two aluminium platens of 0.50 mm deep groves to hold the plastic cement mass and the distance between the two platens was 1.0 mm. The WT and ST were calculated by the changes in the oscillatory motion of the lower platen recorded on a software programme (RDP Electronics Limited Wolverhampton, UK) using an excel chart and were determined by calculating the time taken to reach 95% and 5% of the initial (maximum) amplitude of oscillation, respectively [16]. The values reported are the average of three traces of each cement group.

### 2.5. Statistical Analysis

Statistical analysis was performed by using the Minitab 15.0 (Minitab Limited Coventry, UK). Analysis of variance (ANOVA) was conducted with post hoc Tukey's test comparisons (*P* < 0.05) at the associated 95% confidence interval.

### 2.6. Fourier Transform Infrared (FTIR) Spectroscopy

FTIR spectra of the polymer liquids (FL, FL-V, and FL-N) and glass ionomer cement (F-IX and C-V) were obtained on a Nicolet FTIR spectrometer (FT-Roman Module, MGNA-IR 860) equipped with a mid-infrared source using a DTGS detector having a XT-KBr beam-splitter with a Golden Gate Single Reflection Diamond ATR attachment. For each sample 100 scans were recorded with a 4 cm^−1^ resolution in the range of 2500–700 cm^−1^. The setting reaction of F-IX and C-V cements was studied by the series spectra for one hour by placing the plastic mass of cements on Diamond ATR attachment of spectrometer at one minute after the start of mixing. The cement mass was surrounded by a wet tissue after three minutes of the start of the mixing to prevent dislocation by dehydration.

### 2.7. Molecular Weight Measurement

The number-average molecular weight (*M*_*n*_), the weight-average molecular weight (*M*_*w*_), and polydispersity (*M*_*w*_/*M*_*n*_) of Fuji-IX liquid (FL) were measured at Rapra Technology Limited (Shrewsbury, UK) employing gel permeation chromatography (GPC) using a Vicotek TDA 301 (Column Oven and Detector System with associated Pump and Auto-sampler) at 30°C and at a flow rate of 1.0 mL/min. The GPC (size exclusion chromatography) system was calibrated with sodium polyacrylates calibrants. Solution for GPC analysis was prepared by dissolving 50 mg sample in 10 mL of eluent (0.2 M NaNO_3_, 0.01 M NaH_2_PO_4_  pH ~ 7) and was left overnight to dissolve and then was filtered through 0.45 *μ*m PVDF membrane prior to chromatography. The data was analysed using Polymer Laboratories Cirrus software.

### 2.8. Electron Microscopy

A cryoscanning electron microscope (cryo-SEM) Philips XL30 ESEM-FEG (Philips Co., Japan) at 15 keV at a humidity level of 95% was used to study the fractured surface of the cement specimens from three-point bend test. The GIC samples were cryofixed by plunging it into subcooled nitrogen close to the freezing point of nitrogen at −210°C and then the samples were transferred to the cold-stage of the SEM cryopreparation chamber. After sputter coating with gold, the sample was transferred to the SEM chamber, where it remained frozen on cold-stage during imaging, cooled by nitrogen. A Transmission Electron Microscope (TEM) JEOL-1 200EX (80 kV) was used to examine the dispersion and orientation within the polymer matrix of GICs. GICs cement sample was embedded in a cold moulding resin using a bullet shaped polypropylene mould and was ultramicrotomed (Leica Ultracut E ultramicrotome) to a thickness of 80–110 nm at room temperature using a diamond knife. The TEM samples were placed on a Formvar-coated 200 mesh copper grid. The contrast of the samples was sufficient to permit electron micrograph imaging without staining.

## 3. Results

### 3.1. Mechanical Properties of Cements

The mechanical test results of CS, DTS, FS, and *E*_*f*_ of cements at different storage time are presented in Table 2. Generally, an increase (*P* < 0.05) in the CS values of cements was observed with aging of cements. However, the dispersion of 2.0 wt% PGV nanoclay and 2.0 wt% PGN nanoclay does not show a significant difference (*P* > 0.05) in the CS of cements at 1 hour and 1 month storage. For CS, C-V has the higher mean value at 1 day (137 MPa) and C-N has lowest mean value at 1 day (107 MPa); both values were statistically significant (*P* < 0.05). The mean results of DTS of the cements formed after dispersion of nanoclay at three storage intervals do not show any significant difference (*P* > 0.05) compared to F-IX when data was analysed by one-way ANOVA and post hoc Tukey's test. The cements stored for 1 month have higher DTS than cement stored for 1 day (*P* < 0.01). The FS calculated by the three-point bend test for C-N at 1 hour (30 MPa) and 1 month (28 MPa) storage time was statistically significant (*P* < 0.05) compared to F-IX (25 MPa and 20 MPa). The average FS values of F-IX cement were better at 1 day (30 MPa) and do not show any significant difference (*P* > 0.05) compared to C-V and C-N. Similarly, *E*_*f*_ of cement formed after the nanoclays dispersion (C-V and C-N) at 1 month storage showed no significant difference (*P* > 0.05) compared to the F-IX control group.

### 3.2. Working Time and Setting Time

The results of the working time measurements demonstrated increase (*P* < 0.05) in working time for C-N and similar working time for C-V cements compared to F-IX. The addition of nanoclay resulted in increased (*P* < 0.05) setting time for C-N and C-V, 6.55 (0.10) minute and 6.50 (0.25) minute, respectively (Table 2).

### 3.3. Fourier Transform Infrared (FTIR) Spectroscopy

**Figure 2:**
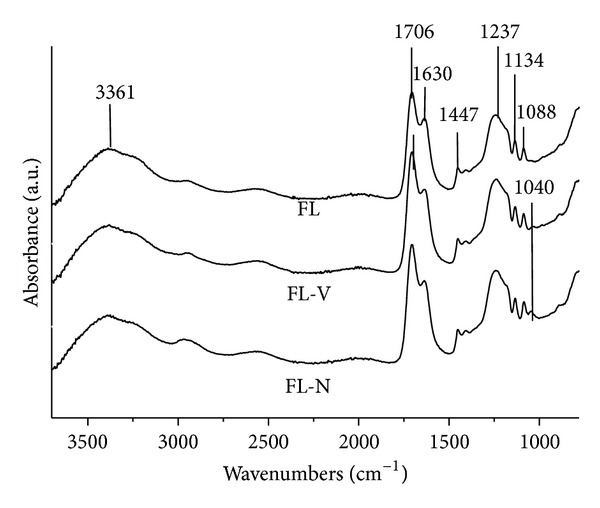
FTIR spectra of Fuji-IX liquid (FL) and solutions formed after dispersion of 2 wt% PGV and PGN nanoclays in FL.

**Figure 3:**
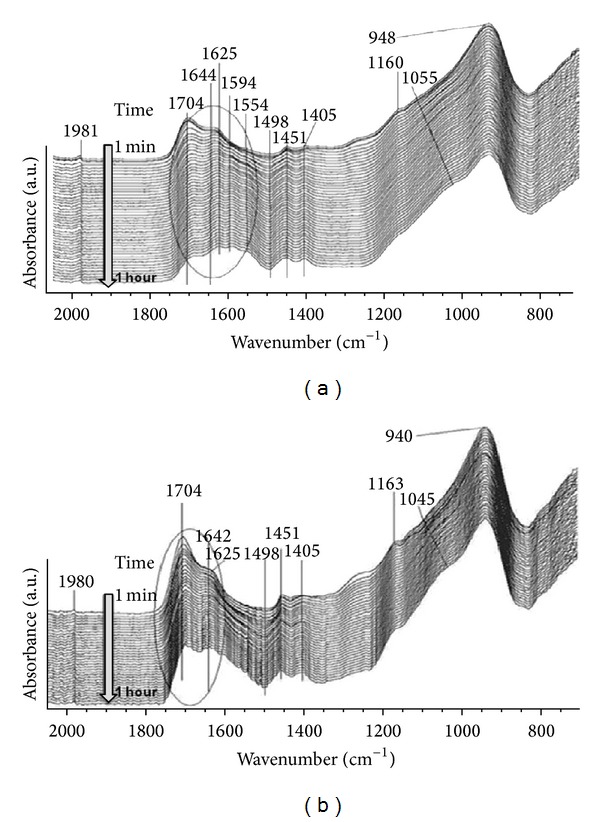
Real time FTIR analysis of the setting reaction of F-IX cement (a) and C-V cement (b) at different time intervals for one hour.

FTIR spectra of Fuji-IX liquid (FL) and the solutions prepared after the dispersion of 2 wt% of PGV and PGN nanoclay (FL-V and FL-N) are shown in Figure 2.  Table 3 gives the measured wavenumbers and their correlation with known vibration frequencies. No obvious peak shift was observed in FL after dispersion of nanoclay. The peak at 1706 cm^−1^ attributed to the C=O stretching vibrations in the carboxylic group which is lost when it neutralised, whereas the peak at 1630 cm^−1^ was associated with –OH bending vibrations of carboxyl group [17]. The peaks at 1088 cm^−1^ and 1134 cm^−1^ suggested the presence of tartaric acid in FL [17]. In the spectrum of FL-V and FL-N, the presence of a weak peak at 1040 cm^−1^ was attributed to the Si–O stretching mode in PGV and PGN nanoclay. The series spectra of glass ionomer cement (F-IX and C-V) at different time interval for 1 hour are shown in Figure 3 and peak assignments are given in Table 3. The weak peak at 1160 cm^−1^ can be assigned to the C–OH stretching vibration of tartaric acid in F-IX. This band decreased in intensity and moved to lower wavenumber at 1055 cm^−1^. The strong band at 1405 to 1451 cm^−1^ was due to the formation of calcium and aluminium polysalts which increased in intensity and became prominent after one hour [17–19]. At one hour after mixing, the symmetric and asymmetric stretching vibrations of aluminium polyacrylate were present at approximately 1450 and 1625 cm^−1^, respectively (Figure 3(a)). The peak around 1644 cm^−1^ was assigned to water sorption but an increase in this absorbance band was small in comparison to changes due to acid-base reaction in the similar band [18]. A strong absorbance band at 948 cm^−1^ due to the stretching vibrations of Si–OH was present continuously over the time periods studied. The asymmetric stretching vibrations in Si–O that usually appear between 940 and 1200 cm^−1^ are an indication for silica gel formation by acid degradation of glass powder [20].  Figure 3(b) shows the setting reaction of cements (C-V) and a strong peak at 940 cm^−1^ was associated with the asymmetric stretching vibrations in Si–OH. The bending vibration at 1625 cm^−1^ moved to higher wavenumbers and attributed to the formation of polyacrylate salts whereas, unlike F-IX, the peak at 1594 cm^−1^ was not present. A gradual increase in the peak intensity at 1642 cm^−1^ was observed with cements aging as expected.

### 3.4. Molecular Weight Determination

**Figure 4:**
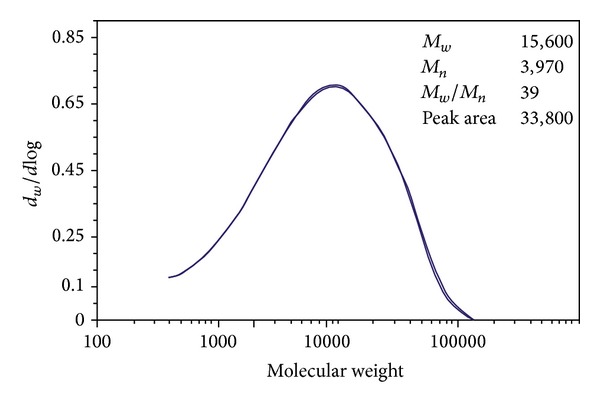
GPC plot showing the molecular weight distribution of FL.

Figure 4 shows an overlay of the computed molecular weight distribution of duplicate run. The polymer solution of Fuji-IX liquid (FL) has a peak in the range of 15,600. The polymer content was calculated from the refractive index detector response. The refractive index chromatogram peak area for the FL was 33,800 indicating the amount of polymer concentration present in the solution. The average *M*_*w*_ was 15,700, *M*_*n*_ was 3,970, and *M*_*w*_/*M*_*n*_ was 39.

### 3.5. Electron Microscopy

**Figure 5:**
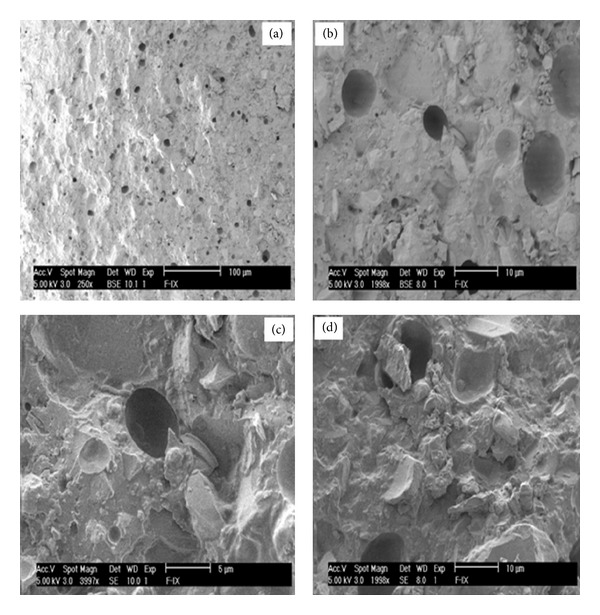
Cryo-SEM micrographs of the fractured surface of GIC at various magnifications demonstrating (a) micrograph at lowest magnification (×250) showing the absence of the cracks and ((b), (c), (d)) micrographs at high magnifications (×1998 and ×3997) indicating the presence of pores, glass particles, and the matrix phase of GIC.

**Figure 6:**
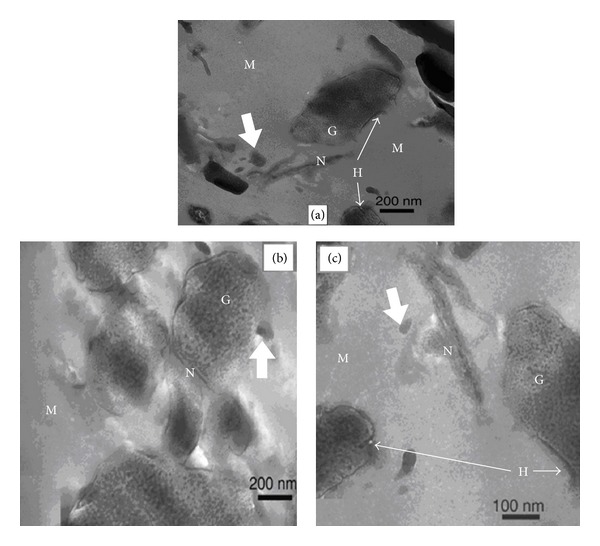
TEM micrographs of Fuji-IX cement after the dispersion of 2% nanoclay in liquid portion (C-N) showing the interaction of nanoclays within GIC system, ((a) and (b)) at lower magnification and (c) at higher magnification indicating remnant glass core (G), nanoclay (N), siliceous hydrogel layers around the periphery of glass filler core (H), cement matrix (M), and fully reacted hydrogel within the matrix (white bold arrow).

The representative cryo-SEM micrographs of the fractured surface of bar-shaped specimens demonstrated few or no microcracks on the surface of glass ionomer cements and the size of the glass particles was measured to be in 5 *μ*m range (Figure 5). Due to the small wt% of nanoclays used, it was difficult to study the dispersion of nanoclays in GIC by scanning electron microscopy. Therefore, transmission electron microscopy (TEM) was employed to study the structure and interaction of nanoclays and GICs. The TEM micromorphological appearance of glass fillers, GIC matrix, and nanoclays is presented in Figure 6. The different phases of glass ionomer cement were readily observed from the TEM micrographs of C-V cement (Fuji-IX with 2 wt% PGV nanoclays). The porous structure of glass filler particles indicated the acid attack of PAA resulting in the formation of siliceous hydrogel layers all around the glass core. The porous nature of glass filler particles was retained within the silica gel layers after depletion of ions from the surface of glass particles [20]. The presence of nanoclays within the GIC matrix was also observed which supported the interaction of PAA with nanoclays. TEM observation confirmed that the layers of nanoclays were mostly exfoliated and dispersed in the matrix of GICs after mixing the PAA liquid constituent containing nanoclay with the aluminosilicate glass powder.

## 4. Discussion

The polymer-grade nanoclay was dispersed in the liquid portion of F-IX to study the effect on cement properties. The dispersion of nanoclays in poly(acrylic acid) solution of F-IX increased the interlayer space of the nanoclay to trap polymer molecules. The possible reaction of PAA with PGV and PGN nanoclays and ion exchange with sodium ions on silicate plates occur during the process of nanoclay dispersion. Polymer chains of PAA can adsorb onto the surface of clay galleries by forming hydrogen bonds or by ion-dipole interaction [11]. The chemical and physical interaction of nanoclay resulted in an ionic bonding at the interface between GIC polymer matrix and the nanoclay which can improve mechanical properties [9]. However, if nanoclays were not fully dispersed and penetrated by polymer chains, then agglomeration of nanoclays would be observed.

Brittle dental restoratives such as GIC have tensile strength values lower than the compressive strength because crack propagation is favoured by tensile forces. The mechanical properties (CS, DTS, FS, and *E*_*f*_) of cements before and after reinforcement with nanoclays showed that all the cements became stronger as they matured at 1 month of storage because it is expected that the setting reaction will continue with time and that the mechanical properties should improve with time. Xie et al. studied the mechanical behaviour of GICs and reported that conventional GICs had higher values of modulus than resin-modified GIC due to the more flexible polymer matrix [21]. In the present work, the modulus of nanoclay clay reinforced cement (C-V and C-N) was higher than control cement, but statistically it was not significant. Therefore, it is suggested that the modulus of GICs after the dispersion of 2 wt% of nanoclays makes these materials suitable for use in load bearing applications as well as for nonload bearing areas. The cements formed after the dispersion of nanoclay generally exhibited higher values or similar value to control group for CS and FS at different storage time but DTS was not statistically different than control group. The significant improvement in the mechanical behaviour of mechanical properties may be restricted by the adjustment in the powder to liquid ratio of the GIC systems after dispersion and the processing technique of nanoclays dispersion in PAA solution. Therefore, it is possible that the graft polymerization of poly(acrylic acid) on the surface of nanoclay would improve the strength of GIC system [9]. The results of the WT and ST depicted a small increase in both the WT and ST of glass ionomer cements prepared after nanoclay dispersion compared to the control group. The dental cement should have a long working time after which it should set rapidly to meet the clinical requirement in oral cavity without disturbing the inherited properties of the materials. The slight deflection in the results to the higher values of WT and ST may be due to the room temperature used during the experiments instead of 37°C. However, such variations in the results may be accepted due to the nature of Wilson's rheometers which is largely dependent upon the elastic tension and the physical properties of the different spring coils used in oscillating rheometers. Previous studies showed the effect of temperature on the working and setting characteristics of cements and an increase in WT and ST at lower temperatures (8°C) [16].

In the F-IX liquid the concentration of PAA in water estimated from refractive index chromatogram peak area was 33 wt % and the *M*_*w*_ of FL that was measured by GPC at RAPRA was in the range of 15.000. It is expected, however, that the molecular weight of PAA of a conventional GIC would have an effect on the nanoclays exfoliation and specifically on the interlayer spacing as the number of polymer entanglements has an effect on the mobility of the chains within the interlayer spacing. This cannot be confirmed by the current study as the effect of PAA molecular weight on the dispersion of nanoclays was not investigated. Kirwan et al. reported that at low pH the attachment of polycarboxylate molecules on hematite surface was not dependent on the chain length [22]. One of the reasons for this could be the difference in the molecular weight as well as the concentration of the polymer in the solution. In order to elucidate the effect of the molecular weight and polymer concentration on the dispersion and exfoliation of nanoclays, a more systematic study should be carried out.

The conventional GIC sets by means of an acid-base reaction between an aqueous PAA solution and a fluoroaluminosilicate glass. It is, therefore, important to study the setting reaction of GICs in order to understand what exactly controls the properties of GICs at the molecular level. During the setting reaction of GICs, poly-acrylate-salt units and tartrate-salt units appear at different wavenumbers making it possible to monitor the activity of various metal cations. The vibration bands from calcium acrylate and aluminium acrylate appear due to the carboxylate stretching vibration [19]. However, FTIR spectroscopy is only suitable for semiquantitative analysis, since the loss of the carbonyl group absorption band during the neutralization overlaps with the formation of the asymmetric COO^−^ salt band and the absorption bands of the polycarboxylic acid overlap with the strong absorption bands of water at 1642 and 1705 cm^−1^ [19]. The FTIR spectra of the FL-N and FL-V were not dissimilar from the spectrum of Fuji-IX liquid. The effects of acid treatment on the structural modification of nanoclays were reported by Madejová et al. which suggested the successive release of the central atoms from the octahedral layer and the release of Al from the Si tetrahedral sheets [23]. In the case of PAA which is considered a weak acid, it can be suggested that the protons from the –COOH groups enter the nanoclay layers and attack the structural –OH groups resulting in dehydroxylation of nanoplates connected with the successive release of the central atoms. This can be demonstrated by observing the changes in the characteristic absorption bands attributed to the vibration of –OH groups and octahedral cations (1642 cm^−1^ and 940 cm^−1^), respectively. The setting reaction of GIC involves the neutralisation of PAA by the glass powder, which is associated with the formation of calcium and aluminium salt complexes. The acid neutralisation extent can be determined from the absorbance changes at 1704 and 1555 cm^−1^ [17]. In comparison to F-IX cement, the real time spectra of C-V show that the peak at 1625 cm^−1^ moved to higher wavenumbers and there was an absence of peak at 1594 cm^−1^ unlike F-IX spectra after one hour from the start of cement mixing. Additionally, the presence of the strong absorbance band at 948 cm^−1^ in both cements was due to the stretching vibrations of Si–OH of the glass powder and no significant changes were found in this band over the duration of one hour. In the F-IX real time FTIR spectrum it is clear that Al- and Ca-tartrates form almost at the same time. However, often it is difficult to differentiate between Ca-poly-acrylates and Al-poly-acrylates resulting in lack of strong evidence that Ca-poly-acrylates form first. In addition, MAS-NMR spectroscopy studies have shown that octahedral hydrated Al^3+^ (sixfold coordinated aluminium Al(VI)) the type of Al^3+^ that forms Al-poly-acrylates forms already at very early stage during the setting reaction [24, 25]. The setting reaction of GICs based on three different fluoroaluminosilicate glass compositions (LG125, ART10, and LG26Sr) using ^27^Al MAS-NMR spectroscopy was recently studied by Zainuddin et al. [26] and it was reported that the setting reaction depended strongly on the glass composition used to form the cement. Specifically, when the glass was rich in phosphorus, the presence of Al–O–P species had an influence on the dissolution of the glass during the acid attack and consequently an effect on the ion release. In the case of the Sr substituted glass, the setting reaction was completed after one day, whereas the setting reaction continued up to 1 year in the case of glass having low phosphorus contents. Furthermore, they reported the presence of Al(VI) with obvious conversion of Al(IV) to Al(VI) at two minutes after the start of the GIC setting reaction. Considering all the above, it is clear that there is no strong evidence to support the idea that Ca-poly-acrylates form earlier than Al-poly-acrylates and Ca and Al compete equally to crosslink the polyacrylic acid chains during the cement formation. Nonetheless, it was expected that the FTIR analysis would give more information on the role of nanoclays in the setting reaction; however, the spectra of cements that contained nanoclays did not give a better insight.

The SEM observation of GICs revealed that the fractured surface of cement specimens consists of both large and small glass particles which can readily be distinguished from the polymer matrix. The cracks formations on GIC surface were avoided by the use of cryo-SEM. Cryo-SEM micrographs did not show the cracks over the surface of GIC samples compared to conventional SEM. Such crack formations during SEM observations are due to the dehydration of cements under high vacuum conditions. Figure 5 shows that glass particles were embedded in the cement matrix and some of the glass particles are also observed on the surface of cements dislodged during the three-point test. Although the average size of glass particles was measured approximately 5 *μ*m in the micrograph of the Fuji-IX cement, there is a possibility that much smaller glass particles may also be present. Transmission electron microscopy (TEM) showed that the cement-forming reaction resulted in the dissolution of the glass to form an amorphous gel around the glass particle. The mesoporous appearance of glass particle etched with PAA and its association with the nanoclay can be observed in Figure 6. Tay et al. described the porous structure of glass filler particles in ChemFlex (GIC) as “seed-like” inclusions and reported that seed-like inclusions were retained within the silica gel layer after the depletion of ions from the surfaces of glass particles in conventional glass ionomer cements [27]. They observed a 150–300 nm thick siliceous hydrogel phase around the glass core and suggested that smaller glass particles reacted completely with PAA forming siliceous-hydrogel rich phases within the cement matrix. On the other hand, Barry et al. suggested that the porous glass filler inclusions present in conventional GIC formulations represent segregated regions of a fluoride-rich phase in certain reactive glass compositions [28]. Extending the explanations of Tay et al. to our work, the presence of siliceous hydrogel phases surrounding the remnant glass core was observed in the TEM micrographs of nanoclay reinforced GIC (C-N). The thickness of this phase seems to be only a few hundred nanometers. The smaller particles forming fully reacted hydrogel phases (core glass has fully reacted) are also shown in Figure 6 (white bold arrow). The TEM of GICs reported in the present study confirmed the presence of porous glass filler particles and our findings are in good agreement with the abovementioned studies. Moreover, the presence of electron-dense (dark gray) zones in the majority of the polymer matrix (M) in Figure 5 was probably due to the formation of a polysalt matrix when metallic ions reacted with PAA. In the setting reaction of GICs, bonds are formed between the polycarboxylic from the PAA matrix and aluminium and/or calcium ions from the glass particles resulting in salt bridges. The unreacted glass filler particles and/or unreacted PAA matrix may constitute a microstructure with inferior mechanical properties. It is therefore assumed that nanoclay reinforcement may reduce the amount of unreacted PAA matrix but it is difficult to comment on such interaction.

## 5. Conclusions

The optimum nanoclay dispersion in the polymer liquid component of the cement is crucial. The dispersion of nanoclays in the liquid portion of F-IX and the reinforcement effect on GICs were determined. Although the dispersion of nanoclays was successfully achieved, a small improvement in the mechanical properties of the GIC systems was observed. The reinforcement of 2 wt% nanoclay generally resulted in improved mechanical behaviour. The incorporation of nanoclay possibly does not compromise the acid-base neutralization reaction with a minimal effect on WT of GIC but provides the reinforcement at nanoscale. Various factors can affect the improvement of the mechanical properties in cements; for example, the adjustment in the P/L ratio of the GIC systems, the polymer concentration, molecular weight of PAA, the size of nanoclays, the nanoclay surface modification method, and the processing technique used are important factors for the stability of the PAA-nanoclay dispersion. It is suggested that the dispersion of nanoclays in lower than 2.0 wt% (1-2.0 wt%) in GICs may potentially produce cements with better mechanical properties.

## Figures and Tables

**Table 1:** Compositions and abbreviations of different experimental groups.

Group	Liquid composition	Powder composition
FL∗	Polyacrylic acid copolymer	Control
FL-V	FL	2 wt% PGV nanoclay
FL-N	FL	2 wt% PGN nanoclay

GIC	GIC liquid	GIC powder

F-IX	FL	Fluoroaluminosilicate glass
C-V	FL-V	F-IX powder
C-N	FL-N	F-IX powder

∗Fuji-IX liquid (FL) is from Fuji-IX GP (F-IX), GC Cooperation, Japan.

**Table 2:** Mechanical properties (CS, DTS, FS, and *E*_*f*_) results of experimental (nanoclay-reinforced GIC) and control group (Fuji-IX) GICs at different storage time. Mean values (standard deviation) of working time and setting time of cements are also presented.

Cement groups	F-IX	C-V	C-N
CS (MPa)			
1 hour	99 (10)^a^	94 (8)^a^	100 (14)^a^
1 day	120 (19)^a^	137 (16)^b^	107 (16)^c^
1 month	124 (19)^a^	122 (17)^a^	130 (26)^a^
DTS (MPa)			
1 hour	12 (2)^a^	10 (2)^a^	10 (4)^a^
1 day	15 (3)^a^	14 (3)^a^	13 (3)^a^
1 month	16 (3)^a^	17 (3)^a^	19 (4)^a^
FS (MPa)			
1 hour	25 (3)^a,c^	20 (4)^b^	30 (3)^c^
1 day	30 (5)^a^	28 (5)^a^	29 (5)^a^
1 month	20 (3)^a^	24 (3)^a,b^	28 (3)^b^
*E*_*f*_ (GPa)			
1 month	11 (2)^a^	13 (2)^a^	12 (3)^a^
Woking time (mins)	4.16 (0.15)^a^	4.15 (0.25)^a^	4.50 (0.20)^b^
Setting time (mins)	6.35 (0.10)^a^	6.55 (0.15)^b^	6.50 (0.25)^b^

Parentheses are standard deviations; different superscript (row) indicates statistically significant different (*P* < 0.05).

**Table 3:** Description of FTIR peaks assignment present in the spectra of Fuji-IX liquid and Fuji-IX cements shown in Figures 2 and 3.

Wave number cm^−1^	Assignment	Reference
GIC		
1705	C=O stretching vibration	[17]
1635	O–H stretching vibration of monomer	[17]
1640	C=C stretching of monomer	[17]
1625	Asymmetric C=O stretching of Al-polycarboxylate	[17–19]
1554	Asymmetric C=O stretching of Ca-polycarboxylate	[17, 19]
1450	Symmetric C=O stretching of Al-polycarboxylate	[17–19]
1405	Symmetric C=O stretching of Ca-polycarboxylate	[17, 19]
1644	Water sorption band in cement	[18]
1640–1590	COO^−^ asymmetric stretching vibration	[17–19]
1450–1405	COO^−^ symmetric stretching vibration	[17–19]
1160, 1050	C–OH stretching vibration	[17, 20]
948	Si–OH stretching vibration	[20]
940–1200	Si–O stretching vibration	[17, 20]
GIC Liquid		
3354	H bonded O–H stretching vibration	[29]
1706	C=O stretching vibration in carbonyl group	[17]
1630	–OH bending vibration in carboxylic group	[17]
1134	Tartaric acid in F-IX	[17, 19]
1080	Tartaric acid in F-IX	[17, 19]
1040	Si–O stretching vibration	[20, 30]
